# Inflammatory Adipokines Decrease Expression of Two High Molecular Weight Isoforms of Tropomyosin Similar to the Change in Type 2 Diabetic Patients

**DOI:** 10.1371/journal.pone.0162908

**Published:** 2016-09-20

**Authors:** Stuart A. Savill, Helen F. Leitch, John N. Harvey, Trevor H. Thomas

**Affiliations:** 1 Betsi Cadwaladr University Health Board, Croesnewydd Road, Wrexham, United Kingdom; 2 School of Medical Sciences, Wrexham Academic Unit, Bangor University, Bangor, United Kingdom; Virgen Macarena University Hospital, School of Medicine, University of Seville, SPAIN

## Abstract

Cardiovascular disease and cancer are increased in Type 2 diabetes. TPM1 and TPM4 genes encode proteins associated with cardiovascular and neoplastic disease. High (HMW) and low (LMW) molecular weight isoforms from TPM1 and TPM4 are altered in several cancer cells and the 3'UTR of TPM1 mRNA is tumour suppressive. Leukocytes influence cardiovascular and neoplastic disease by immunosurveillance for cancer and by chronic inflammation in Type 2 diabetes and cardiovascular disease. The aim was to determine changes in expression of isoforms from TPM1 and TPM4 genes in leukocytes from Type 2 diabetic patients and to use the leukocyte cell line THP1 to identify possible mediators of changes in the patients. Gene expression was determined by RT-qPCR. In diabetes, expression of HMW isoforms from TPM1 were markedly decreased (0.55 v 1.00; p = 0.019) but HMW isoforms from TPM4 were not significantly different (0.76 v 1.00; p = 0.205). Within individual variance in expression of HMW isoforms was very high. The change in expression in HMW isoforms from TPM1 and TPM4 was replicated in THP1 cells treated with 1 ng/ml TNFα (0.10 and 0.12 v 1.00 respectively) or 10 ng/ml IL-1α (0.17 and 0.14 v 1.00 respectively). Increased insulin or glucose concentrations had no substantial effects on TPM1 or TPM4 expression. Decreased TPM1 mRNA resulted in decreases in HMW protein levels. Expression of HMW isoforms from TPM1 is decreased in Type 2 diabetes. This is probably due to increased levels of inflammatory cytokines TNFα and IL-1α in Type 2 diabetes. Lower levels of TPM1 mRNA reduce tumour suppression and could contribute to increased cancer risk in Type 2 diabetes. Decreased HMW tropomyosin isoforms are associated with cancer. Decreased HMW isoforms give rise to cells that are more plastic, motile, invasive and prone to dedifferentiation resulting in leukocytes that are more invasive but less functionally effective.

## Introduction

Tropomyosins are a group of proteins that are a major component of muscle fibres in all types of muscle cell and are a critical component of actin stress fibres in the cytoskeleton of all cells. To fulfil this range of functions tropomyosins are encoded by 4 genes, TPM1, 2, 3 and 4, each of which give rise to multiple protein isoforms by variable exon splicing. These isoforms are either high molecular weight (HMW, 284 amino acids) or low molecular weight (LMW, 248 amino acids). Some tropomyosin isoforms have an important role in diseases where cell dedifferentiation [1], motility and invasion are involved such as cancer and cardiovascular disease.

Actin stress fibres of the cytoskeleton, in which tropomyosin isoforms have a critical function in structure and remodelling [2], determine many aspects of cell development and behaviour. They are important in regulating motility and invasion by leukocytes [3] and in many other aspects of cell membrane function such as recognition by membrane receptors and subsequent intracellular signalling [4]. They are also critical in the development of diseased cells such as in dedifferentiation that is characteristic of neoplastic cells in cancer development [5] and in smooth muscle cells in atheromatous lesions [6].

Protein isoform Tm1, encoded by TPM2, has an important function in cancer development and metastasis and we have shown that TPM2 expression is increased in Type 2 diabetes and by elevated insulin levels (unpublished). However, there is evidence that isoforms from TPM1 and 4 also have a role in disease processes.

HMW isoforms from TPM1 are decreased in squamous cell carcinoma of the oesophagus [7]. In addition, there is evidence that the 3'UTR of TPM1 transcripts can activate the tumour-suppressing RNA-dependent protein kinase R [8] and mediate the effects of the potent tumour promoting microRNA, mir21 [9]. LMW isoforms from TPM4 are increased in breast [10] and uterine cervical carcinoma [11] and also in dedifferentiating smooth muscle cells in atherogenesis [1]. A HMW isoform has been detected in serum from ovarian cancer patients [12]. Changes in disease tissues are important but, in addition, leukocytes play a major part in these diseases in immunosurveillance for cancer [13] and in chronic inflammation that underlies Type 2 diabetes and cardiovascular disease [14]. Adipose tissue macrophages have a central role in the inflammatory process underlying Type 2 diabetes [15]. Furthermore, leukocyte invasion of the vascular endothelium and dedifferentiation of smooth muscle to a phagocytic phenotype are central aspects of vascular disease [16].

Type 2 diabetes is a chronic inflammatory condition [17] in which adipose tissue macrophages have an important function [18]. Thus, in addition to the increased levels of glucose and insulin in Type 2 diabetic patients, there are also significantly higher levels of inflammatory cytokines [19]. Inflammatory cytokines can alter gene expression and have a major role in vascular disease [20] and cancer [21] which are recognized as complications of Type 2 diabetes, where their incidence is greatly increased. These complications also greatly increase the stress on health services as Type 2 diabetes becomes a worldwide epidemic.

We have investigated the expression of tropomyosin isoforms from TPM1 and TPM4 in patients with Type 2 diabetes. Using a cultured leukocyte cell line we have shown that changes associated with diabetes can be caused by inflammatory cytokines rather than directly by the glycaemic environment.

## Methods

### Patient and Control Sample Collection and RNA isolation

Subjects with Type 2 diabetes (n = 45, 49% male) were recruited from hospital diabetes clinics. Age matched normal controls (n = 65, 46% male), with normal blood pressure, BMI of less than 25, no family history of diabetes or hypertension, were recruited from GP surgeries and hospital wards. All were white Caucasian. This study (07/WNo02/2) was approved by the North Wales Central Research Ethics Committee and all participants gave written informed consent. Second samples were collected from 13 Type 2 diabetic patients with a mean of 2 years 8 months between the first and second sample. Blood samples were collected into EDTA tubes and processed within 2 hours. RNA was isolated from 2 ml whole blood using the QIAamp RNA blood mini purification kit (Qiagen) according to manufacturer’s instructions.

### Cell Culture and Treatment

THP1 cells (Public Health England Cultures) were routinely maintained in RPMI1640 medium supplemented with 2 mM glutamine (Life Technologies) and 10% FBS (Life Technologies) at 2-8x10^5^ cells/ml. Cells were differentiated at 5–6.5x10^5^ cells/ml in 4 ml standard growth medium containing 50 ng/ml phorbol 12-myristate 13-acetate (PMA) (Sigma) for 18–24 hours, were then washed twice and incubated for a further 24 hours.

Differentiated cells were treated in serum-free RPMI1640 medium containing 2 mM glutamine, 0.5 ng/ml human recombinant insulin (Life Technologies), 5.5 μg/ml transferrin (Sigma), 6.7 ng/ml selenium (Sigma) and 0.5% albumin (Life Technologies).

Serum-free medium used for low insulin/low glucose treatments contained 0.5 ng/ml insulin and 11.1 mM glucose. Additional conditions were obtained by supplementation to 35.5 mM D-(+)-glucose (Sigma) and 10 μg/ml insulin. After 24 hours incubation, glucose levels reduced to approximately 3–4 mM for low glucose conditions and remained above the detection limit of 19.1 mM for high glucose treatments. Glucose concentrations were measured using an Accu-Chek Aviva NC glucose meter. Cytokines and antibodies used were 1 ng/ml Human Recombinant Tumour Necrosis Factor α (TNFα) (PHC3015L, Life Technologies), 10 ng/ml Human Recombinant Interleukin-1α (IL-1α) (200-LA-002, R&D Systems), 500 ng/ml Human TNFα antibody (AF-210-NA, R&D Systems) and 1 μg/ml Human IL-1α antibody (AF-200-NA, R&D Systems). Cells were incubated for 24 hours in treatment conditions before being washed twice in phosphate buffered saline, detached and pelleted at 200 g. Replicates of the treatments of THP1 cells were performed sixteen times with glucose and insulin, nine times with TNFα and eleven times with IL-1α. RNA was isolated using the RNeasy Plus Mini Purification kit (Qiagen) according to manufacturer’s instructions.

### Real-time PCR

RNA quality/quantity was assessed photometrically by Nanodrop (Thermo Scientific) and agarose gel electrophoresis to determine RNA integrity. RNA was stored below -70°C. Reverse transcription was carried out using the Quantitect Reverse Transcription kit (Qiagen) according to manufacturer’s protocols, including the genomic DNA removal step. cDNA was diluted tenfold and stored below -20°C.

Real time quantitative PCR (RT-qPCR) gene expression analysis was performed according to the MIQE guidelines [22]. Primer pairs (Table 1) were designed using Primer3Plus [23]. To exclude the amplification of genomic DNA, primers were located in different exons or designed to span exon boundaries.

**Table 1:** Primer Sequences.

Isoform	Forward Primer	Reverse Primer
TPM1 HMW	cagatgctgaagctcgacaa	gcacgatccaactcttcc
TPM1 LMW	gggagtagctcgctggag	agctggatgcgtctgttca
TPM4 HMW	ctgaggacaagtgcaagcag	gtccaactcctcctcaacga
TPM4 LMW	cgagaaagctgaaggtgatg	tcctctctcactctcatctgc

Primer sequences used for the amplification of TPM1 and TPM4 high and low molecular weight isoforms.

Melt-curve analysis, gel electrophoresis and DNA sequencing confirmed amplicon specificity and the absence of both interfering primer-dimers and genomic DNA amplification. Amplification efficiencies were 90–110%. RT-qPCR analysis was performed on a Bio-Rad iQ5 using PerfeCTa SYBR Green iQ Supermix (Quanta).

cDNA equivalent to 25 ng total RNA was mixed with 2.5 μl 10x SYBR Green Supermix, 300–500 nM specific forward and reverse primers, volume adjusted to 25 μl. After initial denaturation at 95°C, 40 cycles were performed at 95°C for 15 seconds, 61°C for 45 seconds, followed by melt curve analysis. Two technical replicates were performed for each sample and Ct values averaged. No-template control reactions were included for each primer pair. Reference genes were identified from a panel (Primer Design) analysed by GeNorm and Normfinder software (S1, S2 and S3 Tables) [24]. Normalisation was performed using YWHAZ/CYC1 for human samples and UBE4A/ERCC6/VIPAR for THP1 cells. Normalised relative expression levels were calculated using qBase+ (Biogazelle) ∆∆Ct analysis [25] followed by standardization [26]. P-values were calculated using Student’s t-test or a one-way ANOVA with Tukey’s post hoc test on the log transformed gene expression data given in S4–S8 Tables.

### Western Blotting

Total protein lysates were prepared using CytoBuster Protein Extraction Reagent (Novagen) from differentiated THP1 cells treated with and without TNFα and IL-1α and assayed using the BCA Protein Assay Kit (Novagen) according to the manufacturer’s instructions. 50 μg total protein lysate was mixed with 6X Laemmli buffer (Bioquote), incubated at 95°C for 5 minutes and run on a 13.5% 37.5:1 acrylamide:bisacrylamide 0.1% SDS-PAGE gel. Proteins were transferred onto PVDF membrane using a semi-dry transfer cell (Bio-Rad) in buffer containing 48 mM tris, 39 mM glycine, 0.04% SDS and 20% methanol.

Membrane blocking used SuperBlock T20 (PBS) (ThermoFisher Scientific). Primary antibody was added to 0.2 μg/ml and incubated for 1 hour. After washing in PBS pH 7.4, 0.05% Tween-20, secondary antibody was added to 20 ng/ml in blocking buffer and incubated for 1 hour. Membranes were washed, then incubated for 10 minutes in SuperSignal West Dura Extended Duration Substrate (ThermoFisher Scientific) and visualised using a Fusion FX7 imager (Peqlab). Where required, membranes were stripped in 200 mM glycine, 0.1% SDS, 1% tween-20, pH 2.2.

Primary antibodies used were CGβ6 (University of Iowa Developmental Studies Hybridoma Bank) and anti-β-actin (ab8224, Abcam). The secondary antibody was cross-adsorbed HRP conjugated goat anti-Mouse IgG, IgM (H+L) (31446, ThermoFisher Scientific). Quantification of western blot bands was performed using GelAnalyzer 2010 (www.gelanalyzer.com).

## Results

### Tropomyosin mRNA isoform expression in Type 2 diabetic patients and normal controls

The results (Table 2) show that leukocytes express mRNAs encoding high molecular weight (HMW) and low molecular weight (LMW) tropomyosin isoforms from TPM1 and 4. Where repeat samples were taken from the same individual only the first sample was used to generate the data presented in Table 2.

**Table 2:** Tropomyosin isoform expression in normal controls and patients with Type 2 diabetes.

Gene	Isoform	Group	Mean (95% CI)	P Value
TPM1	HMW	Normal	1.00 (0.63–1.59)	
		Diabetic	0.55 (0.42–0.72)	0.019
	LMW	Normal	1.00 (0.75–1.33)	
		Diabetic	0.94 (0.82–1.07)	0.656
TPM4	HMW	Normal	1.00 (0.71–1.42)	
		Diabetic	0.76 (0.58–0.99)	0.205
	LMW	Normal	1.00 (0.80–1.24)	
		Diabetic	0.81 (0.68–0.97)	0.140

Relative mean (and 95% confidence interval) mRNA expression of high and low molecular weight tropomyosin isoforms in normal control individuals and patients with Type 2 diabetes.

**Fig 1:**
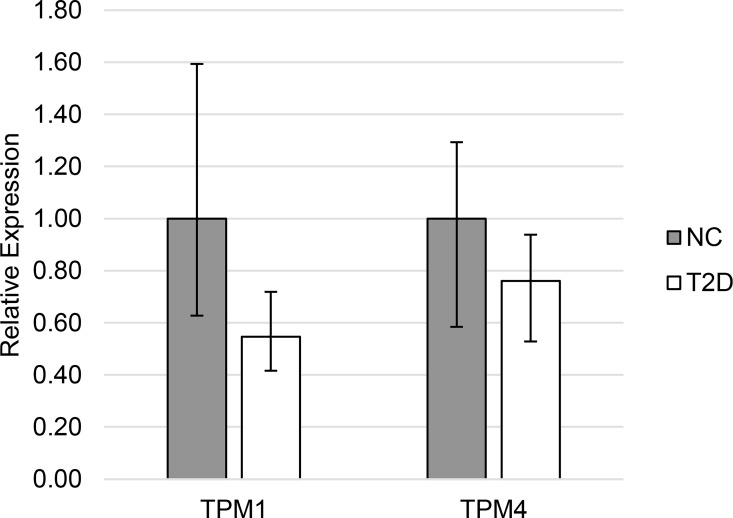
Expression of HMW tropomyosin in Type 2 diabetes. Relative mean (and 95% confidence interval) mRNA expression of HMW tropomyosin isoforms in normal control individuals (NC) and patients with Type 2 diabetes (T2D).

**Fig 2:**
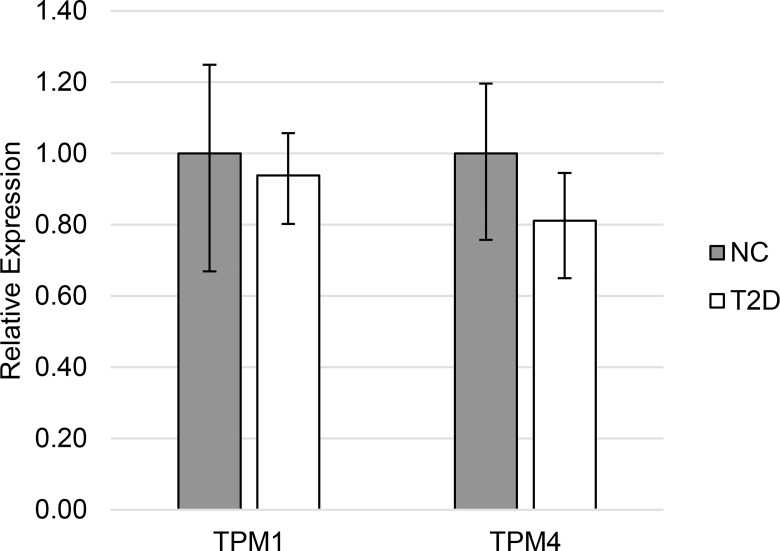
Expression of LMW tropomyosin in Type 2 diabetes. Relative mean (and 95% confidence interval) mRNA expression of LMW tropomyosin isoforms in normal control individuals (NC) and patients with Type 2 diabetes (T2D).

In Type 2 diabetic patients leukocyte mRNAs encoding HMW TPM1 isoforms from exon 1a (Fig 1) were markedly decreased (0.55 v 1.00, p = 0.019). Expression of TPM4 HMW isoforms were not significantly different (0.76 v 1.00, p = 0.21) in leukocytes from Type 2 diabetic patients compared to normal controls. Expression of LMW isoforms from these genes (Fig 2) was not different in diabetes (TPM1: 1.00 v 0.94, p = 0.66; TPM4: 1.00 v 0.81, p = 0.14).

### Variance in tropomyosin mRNA isoform expression

**Fig 3:**
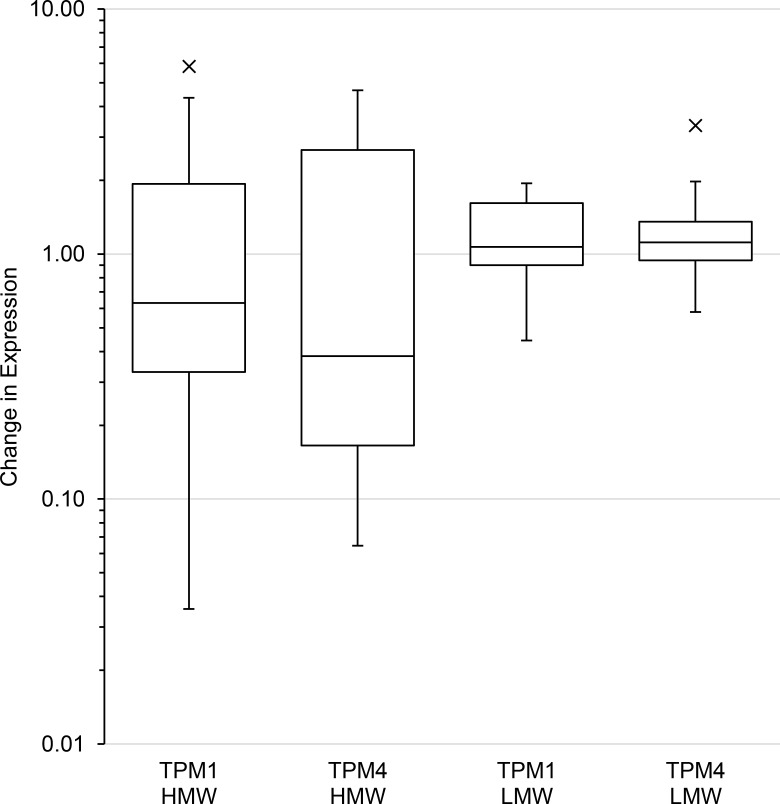
Repeat sampling of individuals with Type 2 diabetes. Box plot showing difference between repeat samplings of patients with Type 2 diabetes. The box represents the interquartile range and the line inside the box indicates the median change in expression. The whiskers are 1.5 times above and below the interquartile range, with outliers individually plotted.

Repeat sampling of individuals showed that the difference in expression within individuals was low relative to assay variance for LMW isoforms from TPM1 and 4 and that the difference was similar between individuals (median difference 1.07, 1.11 respectively; variance of difference 0.23, 0.47 respectively). For TPM1 and TPM4 HMW isoforms the median difference in expression within individuals (0.63, 0.38 respectively) was smaller than for the LMW isoforms. However, the variance in that difference (3.14, 2.39 respectively) was between five- and fourteen-fold greater (Fig 3) indicating that individuals had large changes in expression between repeat samples and that these changes were widely variable. This indicated that the large variance in expression of HMW isoforms in the subject groups was mainly due to fluctuations in expression within each individual. The within individual variance in expression of HMW isoforms was much greater than for LMW isoforms which masked significance of confidence in differences.

### Effect of glycaemic environment on tropomyosin mRNA isoform expression in THP1 cells

Elevated insulin levels had no effect on the expression of HMW or LMW tropomyosin isoforms in THP1 cells although previous studies have shown that insulin does change expression of other genes in these cells [27]. High glucose levels did not have any effect on TPM1 expression but did increase TPM4 LMW isoform expression by 7%. When insulin and glucose levels were both elevated there was a 10% increased expression of HMW isoforms from TPM1 and a 7% increase from TPM4 LMW isoforms (Table 3).

**Table 3:** TPM1 and TPM4 expression in response to glucose and insulin.

Gene	Isoform	Treatment	Mean (95% CI)	P Value (to LILG)
TPM1	HMW	LILG	1.00 (0.95–1.05)	
		HILG	0.95 (0.92–0.98)	0.27
		LIHG	0.97 (0.92–1.02)	0.73
		HIHG	1.10 (1.05–1.15)	0.01
	LMW	LILG	1.00 (0.97–1.03)	
		HILG	1.00 (0.98–1.03)	1.00
		LIHG	1.01 (0.97–1.05)	0.97
		HIHG	1.03 (1–1.07)	0.43
TPM4	HMW	LILG	1.00 (0.9–1.11)	
		HILG	1.07 (0.99–1.16)	0.61
		LIHG	1.01 (0.95–1.08)	1.00
		HIHG	1.15 (1.06–1.26)	0.07
	LMW	LILG	1.00 (0.96–1.04)	
		HILG	1.05 (1.02–1.08)	0.08
		LIHG	1.07 (1.04–1.1)	0.01
		HIHG	1.07 (1.04–1.1)	0.01

Mean tropomyosin isoform expression in THP1 cells in response to elevated concentrations of glucose and insulin.

### Effect of inflammatory cytokines on tropomyosin mRNA isoform expression in THP1 cells

**Fig 4:**
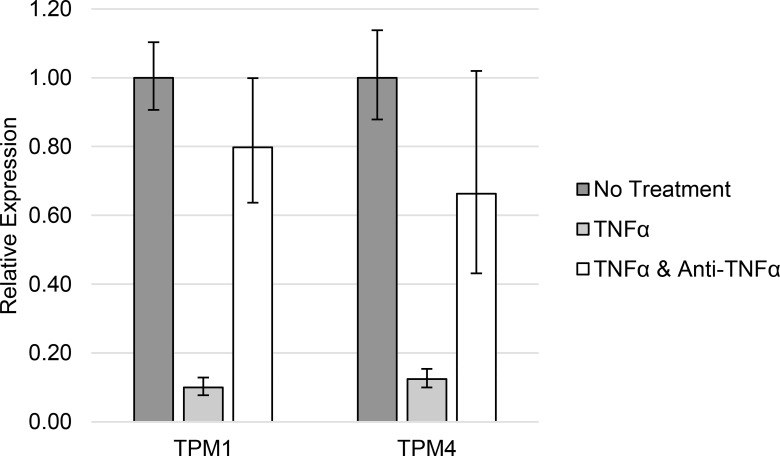
TPM1 and TPM4 expression in response to TNFα. High molecular weight tropomyosin isoform expression in THP1 cells in response to elevated concentrations of TNFα.

**Fig 5:**
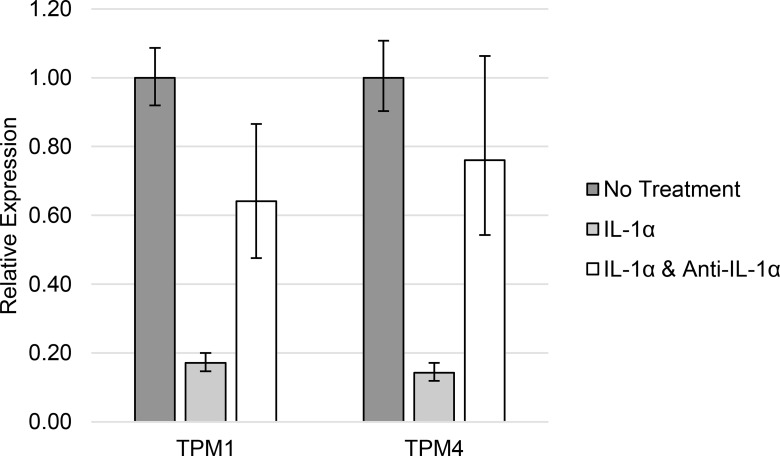
TPM1 and TPM4 expression in response to IL-1α. High molecular weight tropomyosin isoform expression in THP1 cells in response to elevated concentrations of IL-1α.

The difference in TPM1 and TPM4 HMW isoform expression observed between Type 2 diabetic patients and normal controls was replicated in THP1 cells treated with 1 ng/ml TNFα (Table 4 and Fig 4) or 10 ng/ml IL-1α (Table 5 and Fig 5).

**Table 4:** TPM1 and TPM4 expression in response to TNFα.

Gene	No Treatment	1 ng/ml TNFα	1 ng/ml TNFα and 0.5 μg/ml anti-TNF
TPM1	1.00 (0.91–1.10)	0.10 (0.08–0.13)	0.80 (0.64–1.00)
TPM4	1.00 (0.88–1.14)	0.12 (0.10–0.15)	0.66 (0.43–1.02)

High molecular weight tropomyosin isoform expression in THP1 cells in response to elevated concentrations of TNFα.

**Table 5:** TPM1 and TPM4 expression in response to IL-1α.

Gene	No Treatment	10 ng/ml IL-1α	10 ng/ml IL-1α and 1 μg/ml anti-IL-1α
TPM1	1.00 (0.92–1.09)	0.17 (0.15–0.20)	0.64 (0.48–0.87)
TPM4	1.00 (0.90–1.11)	0.14 (0.12–0.17)	0.76 (0.54–1.06)

High molecular weight tropomyosin isoform expression in THP1 cells in response to elevated concentrations of IL-1α.

### Effect of inflammatory cytokines on tropomyosin protein isoform expression in THP1 cells

**Fig 6:**
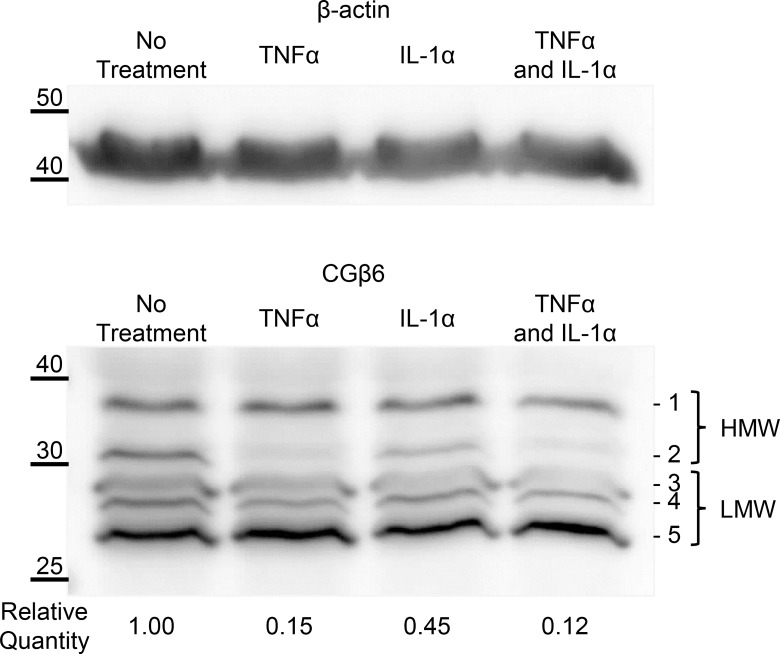
TPM1 protein expression in response to inflammatory cytokines. Western blot showing protein extracts from THP1 cells treated with TNFα and IL-1α. CGβ6 was used to detect both high and low molecular weight TPM1 isoforms. Reported quantitation values are the mean (+/- standard error) of four independent sets of cell treatments each run once andnormalised to total protein loading estimated using Ponceau-S.

Western blotting using the CGβ6 antibody, directed to the TPM1 C-terminus, showed several HMW and LMW protein bands (Fig 6). The decreased mRNA levels due to either TNFα or IL-1α cytokine treatment resulted in corresponding decreases in HMW protein levels from TPM1. No other bands showed consistent changes in protein abundance.

## Discussion

The results of this study strongly indicate that there are decreased levels of expression of HMW isoforms from TPM1 and TPM4 in leukocytes from Type 2 diabetic patients and that these are due to elevated levels of inflammatory cytokines in those patients. There was no evidence that elevated insulin or glucose levels in the patients could cause a decrease in expression from these genes. In fact, on the contrary, elevated insulin or glucose levels caused small but significant increases in expression.

The difference in expression of tropomyosin isoforms in Type 2 diabetic patients could be due to the altered metabolic environment or it could be due to an inherent factor in the diabetic patients. It is difficult to distinguish the role of metabolic factors in the presence of many other influences on gene expression and there were no significant associations between tropomyosin isoform expression and HbA1c or blood glucose levels. However, improvements in glycaemic control not only affect HbA1c and glucose but also many other factors, including inflammatory cytokine levels [28].

It is notable that there is very high variance in the expression of the HMW TPM isoforms that are altered in Type 2 diabetic patients and that are affected by cytokines. It is also notable that this high variance is almost entirely due to variance in expression within individuals with very little additional variance contributed by between individual factors. This was in contrast to the expression of the LMW isoforms from the same genes and also for expression of isoforms from other TPM genes (unpublished observations). The high variability suggests that HMW mRNA isoforms from TPM1 and TPM4 are especially responsive to local conditions and may be involved in modifying the behaviour of the cytoskeleton. This responsiveness occurs at a lower expression level in Type 2 diabetic patients. The high variance impairs statistical confidence that the lower expression of HMW isoforms from TPM1 and TPM4 is associated with diabetes. However, the 45% decrease in TPM1 exceeded conventional statistical significance and even the 24% lower TPM4 had a one in five chance that there is no association of the lower level with diabetic patients.

Therefore, elevated levels of inflammatory cytokines, which are associated with increased risk of both cardiovascular disease [20] and cancer [21], appear to cause marked decreases in expression of HMW isoforms from TPM1 and TPM4. These changes in TPM expression may affect both leukocyte function in respect of immunosurveillance of cancer and atheromatous lesion formation within the vascular endothelium.

## Supporting Information

S1 Table Reference gene selection stability data for patient samples. (XLSX)

S2 Table Reference gene selection stability data in cells treated with glucose and/or insulin. (XLSX)

S3 Table Reference gene selection stability data in cells treated with cytokines. (XLSX)

S4 Table Tropomyosin isoform expression in normal controls and patients with Type 2 diabetes. (XLSX)

S5 Table Repeat sampling of individuals with Type 2 diabetes. (XLSX)

S6 Table TPM1 and TPM4 expression in response to glucose and insulin. (XLSX)

S7 Table TPM1 and TPM4 expression in response to TNFα. (XLSX)

S8 Table TPM1 and TPM4 expression in response to IL-1α. (XLSX)
